# Testing a Model of Consultation-based Reassurance and Back Pain Outcomes With Psychological Risk as Moderator: A Prospective Cohort Study

**DOI:** 10.1097/AJP.0000000000000541

**Published:** 2017-09-28

**Authors:** Nicola Holt, Gemma Mansell, Jonathan C. Hill, Tamar Pincus

**Affiliations:** * Department of Psychology, Royal Holloway, University of London, Egham, Surrey; † Institute of Primary Care and Health Sciences, Keele University, Keele, Staffordshire, UK

**Keywords:** back pain, reassurance, psychological risk, prospective

## Abstract

**Objectives:**

Reassurance is an essential part of treatment for low back pain (LBP), but evidence on effective methods to deliver reassurance remains scarce. The interaction between consultation-based reassurance and patients’ psychological risk is unknown. Our objective was to investigate the relationship between consultation-based reassurance and clinical outcomes at follow-up, in people with and without psychological risk.

**Methods:**

We tested the associations between specific reassurance components (data gathering, relationship building, generic reassurance, and cognitive reassurance), patients’ psychological risk (the presence of depression, anxiety, catastrophizing, or fear-avoidance), and postconsultation outcomes including, satisfaction and enablement, disability, pain, and mood at 3-month follow-up.

**Results:**

Adjusted linear regression models using data from patients who had recently consulted for LBP in primary care (n=142 in 43 practices) indicated that all reassurance components were strongly associated with increased satisfaction, whereas generic reassurance was significantly associated with postconsultation enablement. Generic reassurance was also associated with lower pain at 3 months, whereas cognitive reassurance was associated with increased pain. A significant interaction was observed between generic reassurance and psychological risk for depression at 3 months: high rates of generic reassurance were associated with lower depression in low-risk patients, but with higher rates of depression for high-risk groups.

**Discussion:**

The findings support the hypothesis that different components of reassurance are associated with specific outcomes, and that psychological risk moderates this relationship for depression. Clinicians reassuring behaviors might therefore have the potential to improve outcomes in people with LBP, especially for patients with higher psychological risk profiles.

## Abstract 2

Supplemental Digital Content is available in the text.

Low back pain (LBP) remains highly prevalent and costly worldwide.^1^ Reassurance is an essential part of treatment, with key messages informing patients that serious pathology is not present, the prognosis is usually good, that they should remain active and that further tests are not indicated.^2^ Although reassurance has the potential to enhance self-management and reduce long-term disability, evidence on effective methods to reassure patients with LBP remains scarce and lacking conceptual clarity.

For clinicians practicing patient-centered care, with an emphasis on shared decision-making, delivering effective reassurance is particularly difficult in the context of diagnostic uncertainty, and the limited number of evidence-based treatment options. There is also no clear guidance on the content of reassurance, beyond the message that most patients have a good prognosis and that the presence of serious pathology, in the absence of red flag signs, is very unlikely. At present there is little evidence of clinicians providing either too much, or too little reassurance, or that the type of reassurance given should be tailored according to the patient’s clinical profile.

In a systematic review of prospective cohorts of patients attending primary care,^3^ high levels of patient-perceived affective reassurance (generic positive messages indicating empathy, confidence, and optimism) from their clinician was associated with worse symptom outcome in 3 high-quality studies,^4^–^6^ whereas greater perceived cognitive reassurance (delivering information about etiology, prognosis, and treatment) was linked to improved clinical outcomes in 4 high-quality^4^,^5^,^7^ and 3 lower-quality studies.^8^–^10^

Subsequent studies in LBP^11^,^12^ suggest that affective reassurance includes both implicit and explicit behaviors (Fig. 1). Information-eliciting and relationship-building behaviors provide implicit reassurance, which aims to establish trust, elicit patients’ concerns, and convey the impression that the practitioner cares and is listening,^13^ whereas explicit affective reassurance includes generic statements (you should not be worried about anything serious). Implicit reassurance is believed to be a precursor to the uptake of explicit reassurance, through building of trust and rapport.^12^ Cognitive reassurance is always explicit (Here is what I think is going on, and what I propose we do about it). This distinction allows testing of (1) whether affective reassurance (both implicit and explicit) reduces patients’ ability to take on board information delivered,^5^ resulting in poorer outcomes, or (2) in contrast, whether affective reassurance improves uptake of information through forging trust between practitioner and patients. A model of reassurance^14^ based on the evidence from studies of persuasion argues that affective reassurance, and especially generic reassurance, will enhance a sense of enablement in the short term, but might actually result in worse outcomes later, as patients have not acquired new information to help control their problem.

**FIGURE 1:**
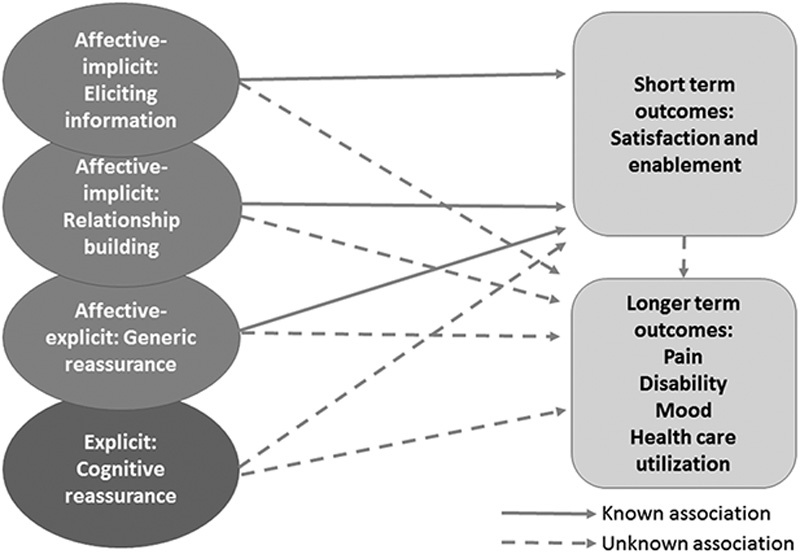
Consultation-based reassurance and patient’ outcomes.

Consultation-based reassurance in this context describes practitioners’ behaviors during the consultation (to differentiate from handing out written material or providing information online) that aim to reduce patients’ concerns. The relationships between consultation-based reassurance and patients’ psychological risk is not known. Psychological factors have been linked both to increased risk of a transition to chronicity and maintenance of chronic pain.^15^–^19^ Differences in patients’ individual characteristics (eg, levels of mood, illness-related cognitions, and expectations) may influence how they respond to different types of reassurance at different points in their illness journey,^20^ and may also affect practitioners’ behaviors. There is a need, therefore, to examine whether psychological risk interacts with (moderates) the impact of reassurance on outcomes.

This study aimed to test the associations between different aspects of patient-perceived reassurance following primary care consultations for LBP and subsequent patient outcomes, overall, and to explore whether there was a significant interaction (moderating effect) in this relationship from patients’ psychological status.

The specific objective was to test how perceived reassurance behaviors relate to LBP outcomes. The following hypotheses were tested:

1. Global hypothesis—reassurance (all components) will be associated with LBP outcomes, after accounting for known predictors.
2. Cognitive reassurance will be associated with improved disability at 3 months, whereas affective reassurance will be associated only with patients’ short-term satisfaction and enablement, but not improved disability in the long term.
3. Psychological risk status will moderate the relationship between reassurance and outcomes.

## METHODS

### Design and Recruitment

This was a prospective observational cohort of patients attending primary care general practice for LBP. In total, 43 general practices in the United Kingdom helped identify patients who had attended for LBP in the previous month (between October 2013 and April 2015). Participants were identified through an electronic search of appropriate Read (diagnostic) codes developed by an independent expert company and carried out by Nurse Practitioners at each practice. Eligible patients were invited to take part in the study by letter, with consent obtained to conduct a postal 3-month follow-up. Ethical approval for this study was granted by the London City and East National Research Ethics Service committee.

The inclusion and exclusion criteria were as follows:

#### Inclusions

- A general practitioner (GP) consultation for LBP within the previous month.
- LBP without radiating leg pain and for whom self-management is indicated (eg, patients were not referred).
- Adult patients (18 y and above).

#### Exclusions

- Red flags for serious potential pathology (such as fracture and inflammatory arthropathies).
- Cancer.
- Cauda equina and ankylosing spondylitis.
- Severe disability or end of life disorders.
- Pregnancy.
- Cognitive impairment or serious mental health problems, which the GP considered could make patients vulnerable and for whom study participation would be detrimental.
- Previous spinal surgery.
- Currently receiving or referred to secondary care (pain-management programs, physiotherapy, etc.) for the same problem.
- Unable to read and speak English.
- Those requiring further investigation or urgent medical attention.

### Measures

**FIGURE 2:**
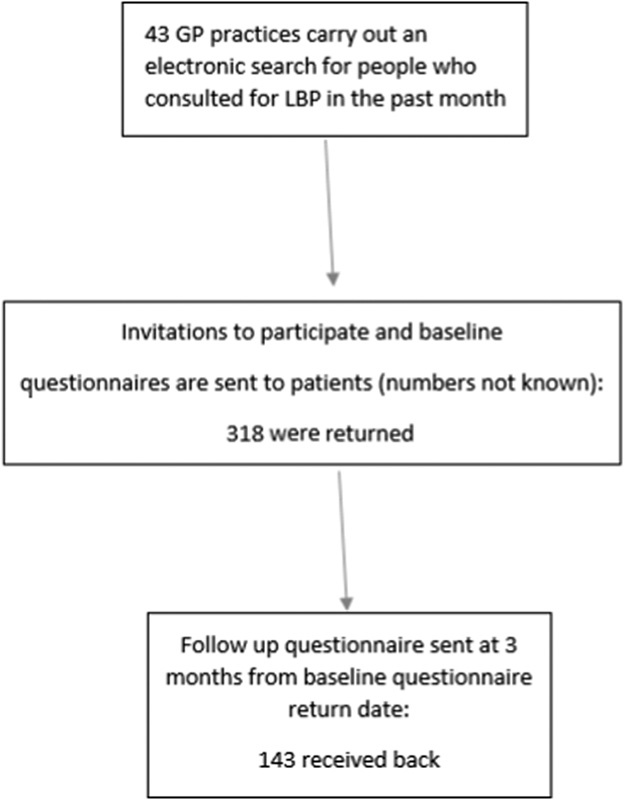
Flow chart of recruitment. LBP indicates low back pain.

Patients received 1 questionnaire by post within a month of their initial consultation (defined as baseline), and another (follow-up) after 3 months (Fig. 2).

Demographic data were collected at baseline. Participants were also asked to report their back pain episode duration using the following options: <1 month; 1 to 3 months; 4 to 6 months; 7 months to 3 years; >3 years. Participants were also asked if this was their first episode of back pain or not.

#### Predictor

*Consultation-based Reassurance Questionnaire (baseline)*. This 12-item questionnaire^11^ measures perceived reassurance specific to consultations for LBP.

This questionnaire was developed through qualitative interviews with patients and quantitative testing, using Rasch modeling in 2 samples from the same population of recent LBP consultations. It includes 4 subscales, each with 3 items: information gathering (eg, to what extent did your doctor … encourage you to voice your concerns regarding your symptoms); relationship building (eg, to what extent did your doctor … show a genuine interest in your problem); generic reassurance (eg, to what extent did your doctor … tell you that everything would be fine); and cognitive reassurance (eg, to what extent did your doctor … check you understood the explanation he/she gave for your symptoms). The response mode to each item describing a practitioner behavior comprises a 0 to 7 Likert scale with the anchors “not at all” to “a great deal.” Each subscale has a range of possible scores from 0 to 21. The authors report that the questionnaire performed with good content validity, consistent responses across groups, and acceptable reliability.

#### Outcomes

*Disability and Pain Intensity (Baseline and 3 mo)*. Functional status (disability) was assessed using the Roland-Morris Disability Questionnaire (RMDQ,^21^), which is a well-validated measure of disability in LBP populations.^22^

Participants were asked to rate their pain intensity in the previous week on the 11-point Pain Numeric Rating Scale.^22^ The Numeric Rating Scale asks patients to rate their pain from 0 (no pain) to 10 (worst possible pain).

*Satisfaction and Enablement (Postconsultation)*. To measure satisfaction, the Consultation Satisfaction Questionnaire (CSQ^23^) was used. The CSQ is a validated 9-item questionnaire in which participants respond to statements about how they felt about the consultation on a 5-point scale from “strongly agree” to “strongly disagree.” The CSQ is scored as a whole, and also provides subscales measuring 4 different aspects of satisfaction: general satisfaction; satisfaction with professional care; satisfaction with the depth of relationship; and satisfaction with perceived time. Enablement was measured with the Patient Enablement Instrument (PEI^24^) which has been validated for use in primary care populations.^25^ The PEI consists of 6 items, rated on a 3-point scale from either “much better” to “same or less” or “much more” to “same or less” which concern patients’ ability to cope with and manage their health/illness. The Cronbach α values in the current population were 0.90 and 0.91 for the CSQ and PEI, respectively, suggested high internal consistency.

*Depression and Anxiety (3 mo)*. The Hospital Anxiety and Depression Scale (HADS^26^) was used to assess participants’ psychological mood outcomes. The HADS is a well-established measure of anxiety and depression that has been validated in both clinical and nonclinical populations, as well as for use in primary care.^27^ It consists of 14 items, 7 of which measure anxiety and the other 7 depression. Scores from the HADS were entered as continuous variables, with a higher score (of a possible 21 each) indicating higher depression or anxiety. Participants are asked to give their responses to the items based on how they have been feeling in the past week. The Cronbach α values in this population were 0.87 and 0.81 for anxiety and depression subscales, respectively, suggesting high internal consistency.

#### Moderator

*Psychosocial Risk (Baseline).* Psychological risk was determined using a modified STarT Back Tool.^28^ The original STarT Back Tool includes 9 items: (1) referred leg pain; (2) comorbid pain; (3) difficulties in walking; (4) difficulties in dressing; (5) fear of physical activity; (6) anxiety; (7) pain catastrophising; (8) depressive mood; and (9) overall impact of pain, with items (5) to (9) making up the psychological subscale. To avoid the risk of incorporation bias, as our primary outcomes were disability and pain, we included only 4 items from the psychological subscale (5 to 8) that specifically relate to psychological risk. We excluded item 9 on the overall impact from pain, because of the ambiguity in this item representing a psychological risk subgroup, rather than indicating a higher level of physical complexity and compromised function.

A positive response to ≥3 of these 4 subscale items was considered sufficient to categorize an individual as psychosocial “high-risk,” whereas a score of ≤2 classified an individual as low-risk. We used this categorization because there is evidence that each of the risk factors independently increases the risk for chronicity.^15^,^18^,^29^ We did not want to use the high-risk cut-off for the whole scale (endorsing all 4 items), because we believed it would miss people who clearly were experiencing psychological difficulties. We therefore relaxed the cut-point to include those who endorsed 3 items.

#### Potential Confounders

Demographic variables used as potential confounders were age, sex, education, sex congruence of patient with clinician length of current pain episode, and number of previous consultation, pain intensity and disability, and psychosocial risk score. Psychosocial risk score was dichotomized into 2 groups, with a score of 0 to 2 indicating low risk and a score of 3 to 4 indicating high risk.

In addition, to control for the effects of repeated consultations, at the 3-month follow-up point participants, reported the number of GP consultations they had had for this episode of LBP, and details of any other physicians they had seen since their consultation.

### Sample Size

We based the power calculation on the a-priori hypothesis that those who perceived greater levels of cognitive reassurance would show a 2.5-point difference on the RMDQ in comparison with those who did not, taking into account 80% power and an α of 5%. We assumed the SD of disability would be equally distributed between the groups at 5.5.^11^ The sample size required was n=76 for each group (receiving reassurance or not). We took into consideration an estimated loss to 3-month follow-up of around 40%, based on a large and relevant study conducted by the World Health Organization,^30^ thus aimed to recruit 315 patients.

### Statistical Analyses

All analyses were performed using SPSS version 21.^31^ Assumptions for normality for each of the variables were examined via the skewness and kurtosis values (where values that deviate from 0 suggest a non-normal distribution) and histograms.

### Hypotheses 1 and 2 ([1] Reassurance Will be Associated With LBP Outcomes; [2] Cognitive Reassurance With Improved Disability, Affective Reassurance Satisfaction and Enablement, But Not Disability)

Linear regression models were performed with all reassurance components entered as a block into the regression as a predictor of each outcome (satisfaction and enablement at the first consultation, and anxiety, depression, pain and disability scores at 3-month follow-up). Both unadjusted and adjusted models were performed, with the adjusted models including confounders entered in 3 blocks before the reassurance components were included in the model. The confounders adjusted for were age, sex, education, sex congruence of patient with physician, length of current pain episode, and number of previous consultation (block 1), pain intensity and disability at baseline (block 2), and dichotomized psychosocial risk score at baseline (block 3). A score of 0 to 2 indicating low risk and a score of 3 to 4 indicating high risk.

### Hypothesis 3 (Psychological Risk Status Moderates the Reassurance and LBP Outcomes Relationship)

Means and SDs of 3-month LBP outcome scores were examined when participants were split into high or low psychosocial risk and low, medium, or high perception of reassurance (reassurance variables were split into tertiles based on score percentages (thirds). Interaction term variables were created that could then be included in linear regression models (reassurance component×psychological risk score). Linear regression models were then run for each of the outcomes listed for objectives 1 and 2, with predictors again entered in blocks (reassurance component [1=low perceived reassurance; 2=medium perceived reassurance; 3=high perceived reassurance] and dichotomized psychological risk score [0=low risk; 1=high risk] [block 1] and the interaction term [block 2]). This was to test the strength of association of the main effect of each variable before the strength of association of the interaction between them. To reduce multiple testing we limited the analysis to 3-month outcomes only (disability, pain, depression, and anxiety).

### Sensitivity Analysis

High levels of missing data at 3-month follow-up (between 45% and 56% for each of the 3-month follow-up variables) and the large numbers of variables controlled for in objectives 1 and 2 meant that the analysis in the adjusted linear regression models could be underpowered. Single imputation was therefore carried out using expectation maximization imputation. This method assumes data are missing at random, which was checked using the Little missing completely at random (MCAR) test^32^ which will be nonsignificant if the data are MCAR. Analyses on the imputed data are given in Appendix 1 (Supplemental Digital Content 1, http://links.lww.com/CJP/A454) as a sensitivity analysis to allow comparison of results between the original and imputed data sets.

## RESULTS

### Sample Participants

In total, 318 participants provided responses to the first questionnaire, of which 34.3% scored as high risk (3 to 4 on the STarT Back tool). Of these, 142 (44.7%) completed the 3-month follow-up questionnaire. Descriptive statistics for the sample can be found in Table 1. There were only 2 factors in which responders and nonresponders differed. Those who completed the follow-up assessment were slightly older (58.1 y, SD 15.0) than those who did not (52.3 y, SD 17.0), and were more likely to have seen a female practitioner (Table 1). The median total scores on each of the reassurance subscales were as follows: information gathering, 15.5 (interquartile range [IQR], 12, 18); relationship-building, 17.0 (IQR, 12, 20); generic reassurance was 12.0 (IQR, 7, 16); and cognitive reassurance, 14.0 (IQR, 10, 18) (Table 2). Kurtosis values for generic reassurance, total enablement score, and 3-month pain intensity score suggested some deviation from normality in these variables. The histograms also suggested that most of the included variables did not follow a normal distribution.

**TABLE 1:**
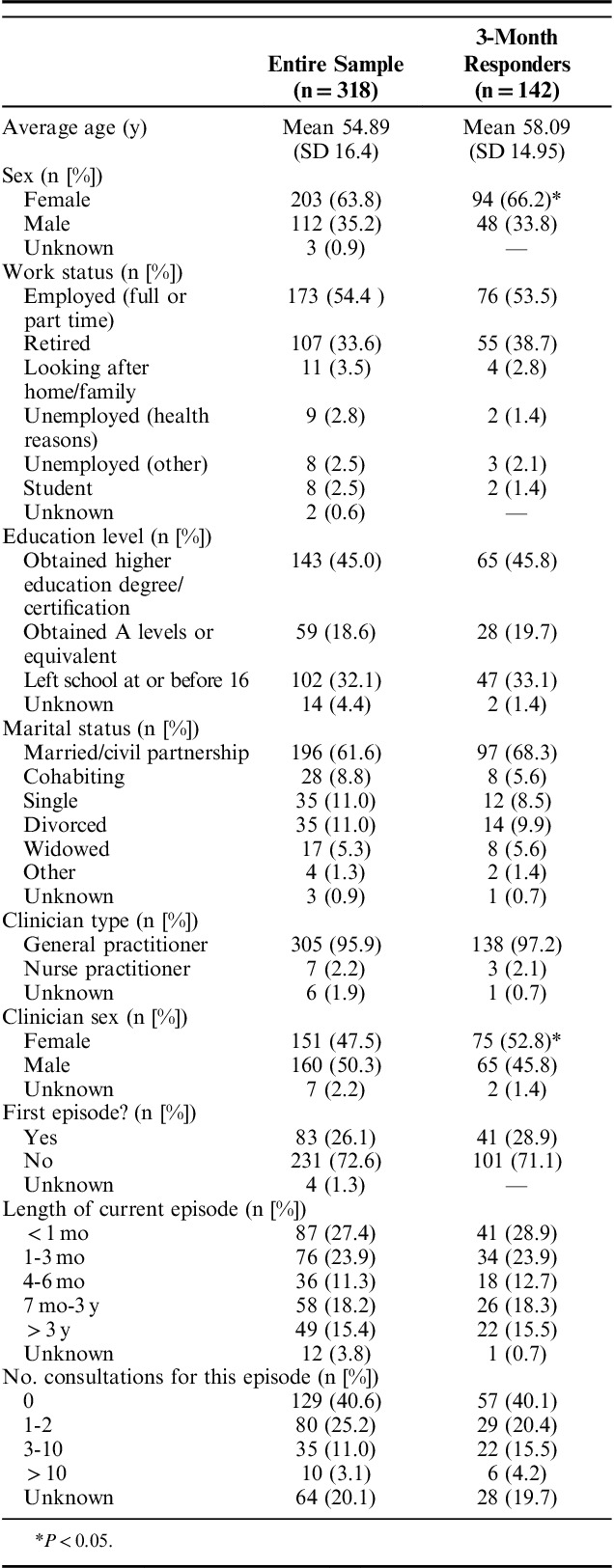
Participant Characteristics

**TABLE 2:**
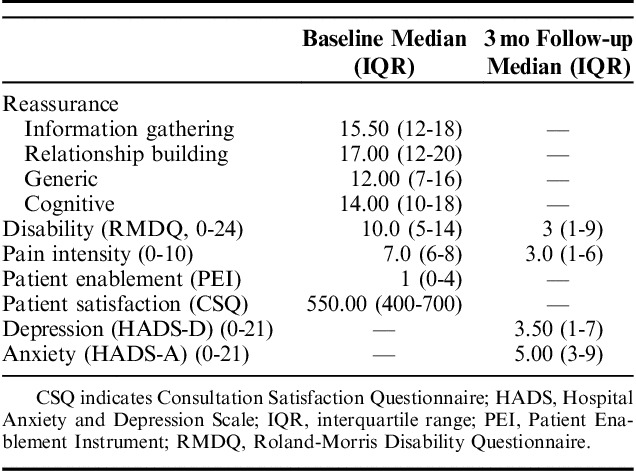
Means and SDs for All Variables Included in Analysis

### Hypotheses 1 and 2 ([1] Reassurance Will be Associated With LBP Outcomes; [2] Cognitive Reassurance With Improved Disability, Affective Reassurance Satisfaction and Enablement, But Not Disability)

Regression parameter estimates from the linear regression analyses are presented in Table 3. For all types of reassurance, greater perceived reassurance was strongly associated with greater patient’ satisfaction; increased generic reassurance was associated with increased enablement, and associated with reduced pain and disability scores at 3-month follow-up in the unadjusted analysis but once adjusted the relationship only remained for increased enablement and reduced pain intensity. Increased cognitive reassurance was associated with increased patient satisfaction and reduced pain scores at 3-month follow-up in both the unadjusted and adjusted analyses. The *R*^2^ change values suggest that when all reassurance components were considered together, they were most important for satisfaction and enablement outcomes, accounting for 69% and 29% of the variance, respectively, in the adjusted analyses, and accounted for very little variance for the 3-month outcomes (between 1% to 8% [adjusted analyses]). Therefore, the hypothesis that reassurance (all components) would be associated with outcomes is partially supported; and the hypothesis that cognitive reassurance only will be associated with disability and that affective reassurance will be associated with patient satisfaction was also partially supported (cognitive reassurance was not associated with disability outcome and while information gathering and relationship-building reassurance were associated with increased patient satisfaction, cognitive reassurance was also associated with this outcome). In the adjusted imputed analyses, there were significant associations between relationship-building reassurance and increased enablement, generic reassurance and reduced disability, cognitive reassurance and increased anxiety, and information-gathering reassurance and reduced anxiety. However, the analysis of imputed data yielded very similar amount of variance explained by the models to that found in the original data, although some.

**TABLE 3:**
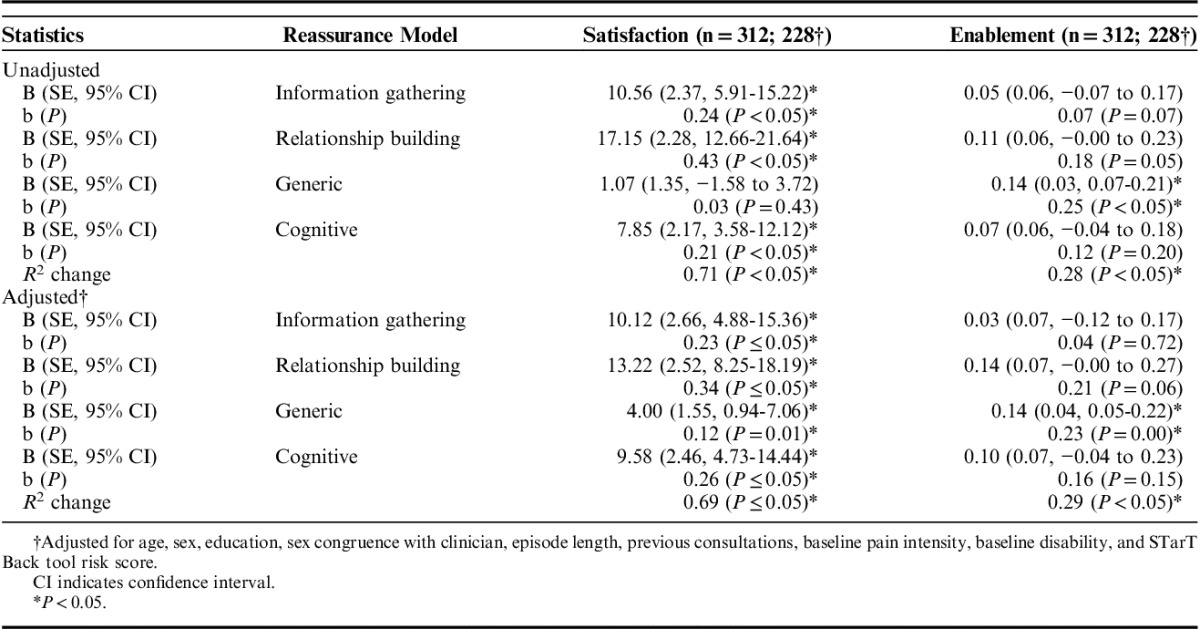
Reassurance as a Predictor of Outcomes Postconsultation and at 3-Month Follow-up; Regression Analysis

Correlations between patient enablement scores at consultation and outcomes at 3 months were run to explore the most likely moderator (enablement) between perceived reassurance and outcomes at 3 months. Enablement was found to have only very weak associations with all outcomes (the Pearson correlation coefficients of −0.04 with pain intensity; −0.09 with disability; −0.07 with depression; and 0.00 with anxiety).

### Hypothesis 3 (Psychological Risk Status Moderates the Reassurance and LBP Outcomes Relationship)

**FIGURE 3:**
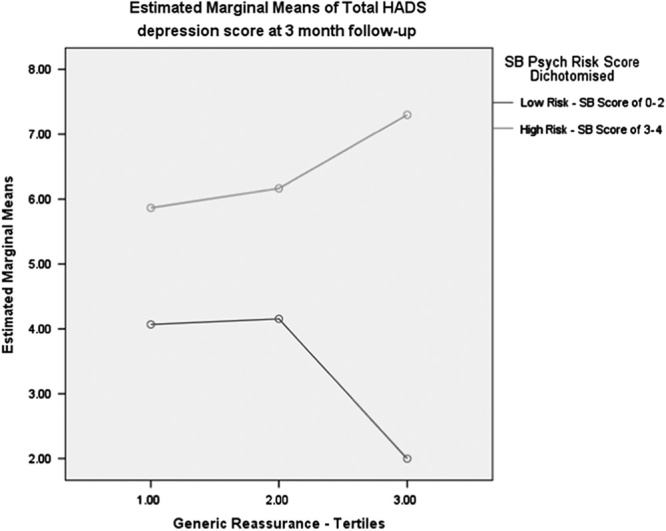
The interaction between reassurance and risk profile on anxiety at follow-up. HADS indicates Hospital Anxiety and Depression Scale. SB indicates StarTBack items.

Table 4 shows the linear regression analyses for Hypothesis 3, which show the interactions between psychosocial risk and type of reassurance. A statistically significant relationship was found for the interaction between generic reassurance and psychological risk for depression score at 3-month follow-up. The change in *R*^2^ values suggested that the interaction between generic reassurance and psychological risk results in a 3% increase in variation explained over and above the main effects of the psychological risk and generic reassurance variables individually. Figure 3 illustrates the interaction: although higher scores in perceived generic reassurance are associated with reductions in depression for low-risk patients, the opposite is found for high-risk patients. For these, the more generic reassurance they perceive to have received, the higher their rates of depression at 3 months. No other statistically significant interactions were found for any outcome. Similarly, analysis of imputed data did not find a statistically significant interaction (Appendix 1, Supplemental Digital Content 1, http://links.lww.com/CJP/A454).

**TABLE 4:**
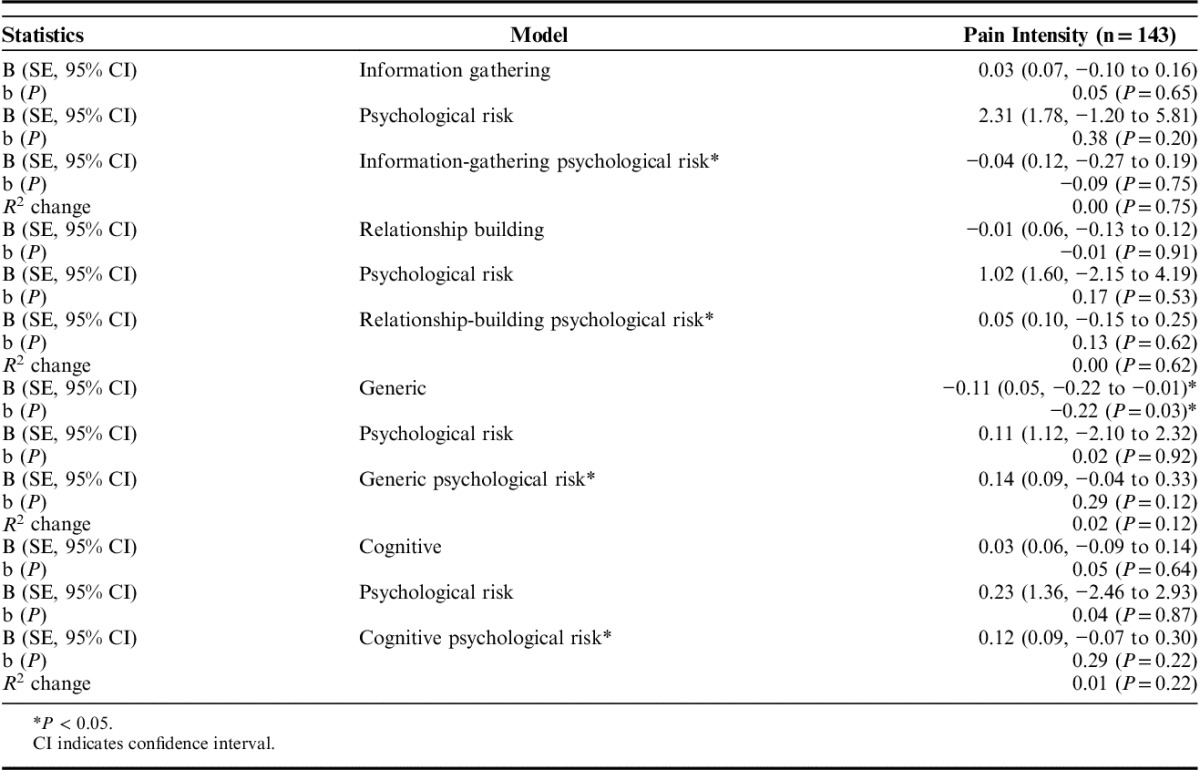
Main Effects and Interactions Between Reassurance and Risk

## DISCUSSION

The findings from this study suggest that reassuring behaviors from GP have an impact on patient’ outcomes. In addition, it seems that the type of reassurance provided could be of importance in people with higher psychological risk. The findings provide support for the hypothesis that patient’ perceptions of reassuring behaviors by their doctors during consultations are associated with some patient’ outcomes, most notably increased patient satisfaction. Furthermore, the findings suggest that the relationship between perceived reassurance and LBP outcomes is moderated by patients’ psychological risk profile, but only in reference to reported depression at 3-month follow-up and not with pain, disability, or anxiety.

Generic reassurance was significantly associated with increased sense of enablement after the consultation, and with a small decrease in reported pain at 3 months. It might reflect doctors’ ability to detect patients who are most likely to recover, but this explanation seems limited, because (1) the association does not extend to disability at 3 months and (2) there is a significant interaction with patients’ risk profile, discussed below. It is surprising that higher rates of reported enablement are not associated with better outcomes, but this is in line with the model of reassurance proposed by Coia and Morley,^14^ who argue that generic reassurance results in immediate reduction of health-related anxiety, but that sense of reassurance is dependent on the clinicians’ presence. When the problem rearises, the patient has acquired no new tools to deal with it. However, it is also possible that the measure of enablement captures a more transient experience, or that the measure of enablement is particularly susceptible to demand characteristics.

Contrary to our prediction, cognitive reassurance was associated with increased pain at follow-up (albeit with a low level of predicted variance). This association may be explained by practitioners utilizing their skills and experience to predict likely prognosis (see above), therefore offering more positive messages to those who they think will improve, and spending more time providing cognitive reassurance to more complex patients, who might recover more slowly. Without a record of what was actually said within the consultation, we can only speculate on the content of the cognitive reassurance received by patients in this sample. That cognitive reassurance was associated with any worse patient outcomes is surprising, as a large body of existing literature suggests that explanations are valued by patients, address their concerns, and help them to recover.^12^,^33^–^35^ It may be the case that the reassurance provided to participants was not sufficient to have a positive effect. Within the limited time available for GP consultations,^36^ this level of intervention might not be possible. There is evidence that GPs tend to stick closely to biomedical explanations, without exploring the psychosocial context of a patient’s problems.^37^,^38^ Thus, patients reported that they received explanations, but we failed to ask them if they agreed with these explanations, or continued to believe that there was a different serious and threatening process going on within their spine.

We note that adequate provision for psychologically at-risk patients may still be wanting in primary care: a large proportion of our cohort were classified as at-risk, and according to recommendations,^39^ should have been referred to multidisciplinary interventions, and therefore excluded from the study. Within the stratified model of care advocated by the developers of the STarT Back tool,^28^ more intensive psychological intervention is recommended for high-risk patients.^40^

### The Interaction Between Risk Profile, Reassurance, and Outcomes

The findings suggest an interaction between generic reassurance and patient’ risk in association with depression at 3 months. Although low-risk patients who received generic messages about likely recovery had the lowest rates of depression, the opposite was found for patients at high risk despite the fact that these patients reported reductions in pain. For these high-risk patients such messages were associated with higher rates of depression, possibly because they failed to address their catastrophic thinking, or, because the reassurance was perceived as being false when the pain did not improve as much they expected or were led to hope.

### Strengths and Limitations

This is the first prospective cohort study to use a validated measure of perceived reassurance for LBP. Previous research has inferred reassurance from patients’ outcomes, for example, see Traeger et al^41^ or measured proximal consultation processes.^3^ The questionnaire utilized in this study has been specifically validated in LBP populations and has been shown to reliably measure patients’ perceptions of reassurance.^11^ In addition, this is the first study to examine the impact of reassurance on different subgroups of LBP patients. This study therefore provides the first step into understanding what can be done to reassure patients with varying psychological risk profiles.

However, the study findings should be viewed with caution due to several limitations. The response rate fell short of the expected 60%, and resulted in possible underpowering to detect interactions for some outcomes. The sensitivity analysis on imputed data did identify some differences between the findings in the original and imputed data, and the Little MCAR test indicated that the data were not missing at random, suggesting there is a potential for bias. Another potential limitation of the study is that participants provided recall up to 1 month following their care visit, and this recall may be confounded by changes in symptoms and other care experiences that occurred within the 1-month period. In addition we did not power the study to test the significance in specific subgroups, to further explain significant interactions, and strongly propose that future research does so. We also note that measures of mood was only taken at the 3-month follow-up. A stronger design would include both these variables at baseline.

The search strategy meant that more complex cases of LBP, which had been referred on to other specialists, were not included. Reassuring behaviors for these patients, therefore, are not represented in this study. In addition, the follow-up period in this study was only 3 months. Future studies should recruit samples that include more complex cases, and measure the long-term effects of reassurance on patients’ outcomes. An ideally designed study would have baseline data collected preconsultation, and postconsultation measures (including reassurance) collected directly after the consultation to avoid recall bias. We could not do so, because of ethical restraints on questioning patients about their reasons for consulting before the consultation, and providing at least 48 hours to consider whether they agreed to take part in the study. We were also unable to measure the number of patients identified for the study: our original design required clinical staff (who due to ethical requirements are the only personnel with access to patients records) to keep and report numbers, but in practice, in busy surgeries, staff were not able to do so reliably.

The reassurance measure used in this study relies on patient self-report, which is based on their perceptions of what happened during a consultation. Although this is valuable information, it may not reflect the consultation reliably. Future research in which patients’ perceptions of reassurance are measured alongside direct observation of consultations will allow testing whether physicians’ attempts at reassurance are, in fact, recognized by (all) patients, and what the implications are when reassurance is not perceived, or is not offered.

We included only 4 items from the validated STarT Back tool psychological subscale, and so chose an mid-point cut-point for classifying patients into high/low-risk subgroups. We note that the single items on the STarT Back represent strongly evident risk factors, and that there is considerable evidence that the more of these are endorsed by patients, the higher the risk for poor prognosis. Although our classification may have lacked sensitivity, it is likely to result in our failure to detect existing associations, rather than in a type 1 error.

### Implications

The findings from this study, in combination with mounting evidence from other studies, suggests that reassuring behaviors from GP could be improved to have impact on patient’ outcomes. Of importance, such behaviors should be studied to improve reassurance in people with higher psychological risk. Current guidelines^2^ that recommend delivery of reassurance fall short of advising on the content, and method of delivery, or tailoring of such reassurance.

A systematic review^42^ of 12 qualitative (n=490) and 8 quantitative (n=3755) studies summarized evidence from studies with LBP patients on their expectations and satisfaction with treatment. The review suggested that patients were mostly dissatisfied with the amount of information provided by their practitioner. The review concludes that practice guidelines should include instructions on *how* to discuss the causes and diagnosis with the patient; however, to date such guidelines have not been produced.

The findings confirm that different reassuring behaviors are associated with different outcomes. Future research is needed to clarify the effect of generic reassurance, especially when offered to patients who present with psychological obstacles to recovery. There is evidence suggesting that these behaviors lead to better patient outcomes.^43^ This study offers the first evidence suggesting that offering such reassurance to more complex patients might actually result in worse outcomes, at least in reference to low mood. Relationship building and appropriate levels of data collection might also be improved, as they were not associated in this study with improved outcomes, although they were strongly associated with patient’ satisfaction. The patient-centeredness movement is built on the notion that physicians should aim to understand a patient’s whole situation and build a therapeutic relationship,^35^ and, indeed, a number of previous studies^44^,^45^ have confirmed that patients value these behaviors. There is evidence that patients with LBP value emotionally reassuring behaviors, particularly interpersonal behaviors that display caring, empathy and warmth; however, the provision of clear explanations was rated as the most reassuring aspect of the consultation.^12^ How to provide clear explanations, and adjust these in the context of uncertainty to match individual patients’ needs remain a challenge, and should form a priority for future research. The current study offers some evidence, but it was underpowered for subgroup analysis for risk, and the findings must therefore be viewed with caution. Future studies should consider subgroups a-priori and plan for sufficient power to test the interactions between psychological risk and reassurance styles. Until such work is carried out the clinical utility of existing studies, including this one, remain limited.

In conclusion, we offer preliminary evidence that current provision of reassurance for people consulting for LBP improves satisfaction and fosters a short-term sense of enablement, but this does not translate into better outcomes at follow-up. In addition, at present, people with psychological risk profiles are not adequately identified or reassured.

## Supplementary Material

SUPPLEMENTARY MATERIAL

Supplemental Digital Content is available for this article. Direct URL citations appear in the printed text and are provided in the HTML and PDF versions of this article on the journal’s Website, www.clinicalpain.com.
