# Supplementary material for: Testing a Model of Consultation-based Reassurance and Back Pain Outcomes With Psychological Risk as Moderator: A Prospective Cohort Study
Source: Clin J Pain. 2017 Sep 28;34(4):339–48. doi: 10.1097/AJP.0000000000000541 (PMC5844580; doi:10.1097/AJP.0000000000000541)
Supplement: SUPPLEMENTARY MATERIAL [file ajp-34-339-s001.docx]

**Appendix 1: Sensitivity analysis using imputed data**

**Table 1: Participant characteristics – Imputed data**

|  | | Entire Sample (*n*=318) | 3-month responders (*n*=318) |
| --- | --- | --- | --- |
| Average Age | | Mean 54.88 (SD 16.30) | - |
| Gender | Female | *n*=206 (64.8%) | - |
|  | Male | *n*=112 (35.2%) | - |
| Work status | Employed (full or part time) | *n*=173 (54.4% ) | - |
|  | Retired | *n*=108 (34.0%) | - |
|  | Looking after home/family | *n*=12 (3.8%) | - |
|  | Unemployed (health reasons) | *n*=9 (2.8%) | - |
|  | Unemployed (other) | *n*=8 (2.5%) | - |
|  | Student | *n*=8 (2.5%) | - |
| Education level | Obtained higher education degree/certification | *n*=144 (45.3%) | - |
|  | Obtained A levels or equivalent | *n*=71 (22.3%) | - |
|  | Left school at or before 16 | *n*=103 (32.4%) | - |
| Marital status | Married/civil partnership | *n*=196 (61.6%) | - |
|  | Cohabiting | *n*=31 (9.7%) | - |
|  | Single | *n*=35 (11.0%) | - |
|  | Divorced | *n*=35 (11.0%) | - |
|  | Widowed | *n*=17 (5.3%) | - |
|  | Other | *n*=4 (1.3%) | - |
| Physician type | GP | *n*=311 (97.8%) | - |
|  | Nurse Practitioner | *n*=7 (2.2%) | - |
| Physician gender | Female | *n*=155 (48.7%) | - |
|  | Male | *n*=163 (51.3%) | - |
| First episode? | Yes | *n*=83 (26.1%) | - |
|  | No | *n*=235 (73.9%) | - |
| Length of current episode | <1 month | *n*=87 (27.4%) | - |
|  | 1-3 Months | *n*=79 (24.8%) | - |
|  | 4-6 Months | *n*=40 (12.6%) | - |
|  | 7 Months – 3 Years | *n*=61 (19.2%) | - |
|  | >3 Years | *n*=51 (16.0%) | - |
| Number of consultations for this episode | 1-2 | *n*=267 (84.0%) | - |
|  | 3-10 | *n*=40 (12.6%) | - |
|  | >10 | *n*=11 (3.5%) | - |
| STarT Back score | Low (0-2) | *n*=209 (65.7%) | - |
|  | High (3-4) | *n*=109 (34.3%) | - |

**Table 2: Means and SDs for all variables included in analysis**

|  | | **Baseline Median (IQR)** | **3 month follow-up Median (IQR)** |
| --- | --- | --- | --- |
| Reassurance | Data gathering | 15.50 (12,18) | - |
|  | Relationship building | 17.00 (12,20) | - |
|  | Generic | 12.00 (7,16) | - |
|  | Cognitive | 14.00 (10,18) | - |
| Disability (RMDQ, 0-24) | | 7.0 (6,8) | 4.0 (2,5) |
| Pain intensity (0-10) | | 10.0 (5,14) | 4.0 (1,8) |
| Patient enablement (PEI) | | 1 (0,4) | - |
| Patient satisfaction (CSQ) | | 550.00 (400,700) | - |
| Depression (HADS) (0-21) | | - | 4.00 (2,6) |
| Anxiety (HADS) (0-21) | | - | 6.00 (4,9) |

**Table 2: Reassurance as a predictor of outcomes post consultation and at 3 months follow up; regression analysis – Imputed data**

| **Statistics** | | **Reassurance Model** | **Satisfaction (*n*=318)** | **Enablement (*n*=318)** | **Pain intensity (*n*=318)** | **Disability (*n*=318)** | **Depression (*n*=318)** | **Anxiety (*n*=318)** |
| --- | --- | --- | --- | --- | --- | --- | --- | --- |
| **Unadjusted** | **B (SE, 95% CI)** | **Data gathering** | 9.37 (2.34, 4.78 to 13.96)* | 0.05 (0.06, -0.06 to 0.17) | 0.10 (0.05, -0.00 to 0.20) | 0.27 (0.10, 0.07 to 0.47)* | 0.02 (0.07, -0.12 to 0.16) | -0.08 (0.09, -0.26 to 0.10) |
|  | **b (*p*)** |  | 0.21 (*p*<0.05) | 0.07 (*p*=0.37) | 0.17 (*p*=0.06) | 0.25 (*p*=0.01)* | 0.03 (*p*=0.75) | -0.08 (*p*=0.39) |
|  | **B (SE, 95% CI)** | **Relationship building** | 17.63 (2.32, 13.07 to 22.18)* | 0.11 (0.06, 0.00 to 0.22) | -0.11 (0.05, -0.21 to -0.01)* | -0.11 (0.10, -0.31 to 0.09) | -0.01 (0.07, -0.15 to 0.13) | -0.02 (0.09, -0.20 to 0.16) |
|  | **b (*p*)** |  | 0.44 (*p*<0.05)* | 0.18 (*p*=0.05) | -0.22 (*p*=0.03)* | -0.11 (*p*=0.28) | -0.02 (*p*=0.89) | -0.02 (*p*=0.82) |
|  | **B (SE, 95% CI)** | **Generic** | 1.15 (1.36, -1.54 to 3.83) | 0.14 (0.03, 0.07 to 0.21)* | -0.18 (0.03, -0.24 to -0.12) | -0.28 (0.06, -0.40 to -0.17)* | -0.09 (0.04, -0.17 to -0.01)* | -0.10 (0.05, -0.20 to 0.01) |
|  | **b (*p*)** |  | 0.03 (*p*=0.40) | 0.25 (*p*<0.05)* | -0.40 (*p*<0.05)* | -0.32, (*p*<0.05)* | -0.15 (*p*=0.03)* | -0.13 (*p*=0.06) |
|  | **B (SE, 95% CI)** | **Cognitive** | 8.52 (2.19, 4.21 to 12.83)* | 0.07 (0.05, -0.04 to 0.17) | 0.20 (0.05, 0.10 to 0.29)* | 0.07 (0.10, -0.12 to 0.25) | 0.06 (0.07, -0.07 to 0.19) | 0.18 (0.09, 0.01 to 0.35)* |
|  | **b (*p*)** |  | 0.23 (*p*<0.05)* | 0.11 (*p*=0.23) | 0.41 (*p*<0.05)* | 0.07 (*p*=0.49) | 0.10 (*p*=0.35) | 0.22 (*p*=0.03)* |
|  | ***R-square change*** |  | *0.70 (p<0.05)* | *0.28 (p<0.05)* | *0.15 (p<0.05)** | *0.09 (p<0.05)* | *0.02 (p=0.28)* | *0.02 (p=0.18)* |
| **Adjusted^#^** | **B (SE, 95% CI)** | **Data gathering** | 9.73 (2.36, 5.09 to 14.37)* | 0.07 (0.06, -0.04 to 0.19) | 0.03 (0.04, -0.05 to 0.11) | 0.08 (0.07, -0.06 to 0.22) | -0.09 (0.06, -0.20 to 0.03) | -0.16 (0.07, -0.30 to -0.02)* |
|  | **b (*p*)** |  | 0.22 (*p*<0.05)* | 0.11 (*p*=0.20) | 0.06 (*p*=0.41) | 0.07 (*p*=0.26) | -0.12 (*p*=0.13) | -0.17 (*p*=0.02)* |
|  | **B (SE, 95% CI)** | **Relationship building** | 16.34 (2.37, 11.66 to 21.01)* | 0.12 (0.06, 0.01 to 0.23)* | -0.06 (0.04, -0.14 to 0.02) | 0.04 (0.07, -0.09 to 0.18) | 0.11 (0.06, -0.01 to 0.22) | -0.14 (0.07, -0.00 to 0.27) |
|  | **b (*p*)** |  | 0.41 (*p*<0.05)* | 0.19 (*p*=0.04)* | -0.12 (*p*=0.14) | 0.04 (*p*=0.53) | 0.16 (*p*=0.06) | 0.16 (*p*=0.06) |
|  | **B (SE, 95% CI)** | **Generic** | 1.16 (1.42, -1.65 to 3.96) | 0.12 (0.04, 0.05 to 0.19)* | -0.11 (0.03, -0.16 to -0.06)* | -0.11 (0.04, -0.19 to -0.03)* | -0.02 (0.03, -0.09 to 0.04) | -0.04 (0.04, -0.12 to 0.05) |
|  | **b (*p*)** |  | 0.03 (*p*=0.42) | 0.22 (*p*<0.05)* | -0.24 (*p*<0.05)* | -0.13 (*p*=0.01)* | -0.04 (*p*=0.51) | -0.05 (*p*=0.41) |
|  | **B (SE, 95% CI)** | **Cognitive** | 9.03 (2.20, 4.70 to 13.36)* | 0.05 (0.05, -0.05 to 0.16) | 0.18 (0.04, 0.10 to 0.25)* | 0.03 (0.07, -0.10 to 0.15) | 0.04 (0.05, -0.07 to 0.14) | 0.14 (0.07, 0.01 to 0.27)* |
|  | **b (*p*)** |  | 0.24 (*p*<0.05)* | 0.08 (*p*=0.33) | 0.36 (*p*<0.05)* | 0.03 (*p*=0.70) | 0.06 (*p*=0.51) | 0.17 (*p*=0.03)* |
|  | ***R-square change*** |  | *0.66 (p<0.05)** | *0.26 (p<0.05)** | *0.07 (p<0.05)** | *0.01 (p=0.04)** | *0.01 (p=0.14)* | *0.03 (p=0.00)** |

**p<0.05; ^#^adjusted for age, gender, education, gender congruence with clinician, episode length, previous consultations, baseline pain intensity, baseline disability and STarT Back tool risk score; Red highlight=difference in statistical significance from data reported in paper*

***Table 3: Main effects and interactions between Reassurance and Risk – Imputed data***

| **Statistics** | | **Model** | **Pain intensity**  **(*n*=318)** | **Disability**  **(*n*=318)** | **Depression**  **(*n*=318)** | **Anxiety**  **(*n*=318)** |
| --- | --- | --- | --- | --- | --- | --- |
|  | **B (SE, 95% CI)** | **Data Gathering** | 0.08 (0.04, 0.01 to 0.15)* | 0.09 (0.07, -0.04 to 0.21) | 0.03 (0.04, -0.05 to 0.12) | 0.04 (0.06, -0.07 to 0.16) |
|  | **b (*p*)** |  | 0.14 (*p*=0.04)* | 0.08 (*p*=0.19) | 0.05 (*p*=0.46) | 0.05 (*p*=0.45) |
|  | **B (SE, 95% CI)** | **Psychological Risk** | 1.31 (0.99, -0.65 to 3.26) | 3.79 (1.78, 0.29 to 7.29) | 2.71 (1.20, 0.34 to 5.07)* | 4.28 (1.56, 1.21 to 7.35)* |
|  | **b (*p*)** |  | 0.25 (*p*=0.19) | 0.38 (*p*=0.03) | 0.41 (*p*=0.03)* | 0.50 (*p*=0.01)* |
|  | **B (SE, 95% CI)** | **Data gathering * Psychological risk** | 0.03 (0.07, -0.10 to 0.16) | 0.06 (0.12, -0.17 to 0.29) | 0.02 (0.08, -0.14 to 0.17) | -0.03 (0.10, -0.23 to 0.18) |
|  | **b (*p*)** |  | 0.09 (*p*=0.64) | 0.10 (*p*=0.60) | 0.04 (*p*=0.83) | -0.05 (*p*=0.80) |
|  | ***R-square change*** |  | *0.00 (p=0.64)* | *0.00 (p=0.60)* | *0.00 (p=0.83)* | *0.00 (p=0.80)* |
|  | **B (SE, 95% CI)** | **Relationship building** | 0.02 (0.04, -0.05 to 0.09) | 0.01 (0.06, -0.12 to 0.14) | 0.06 (0.04, -0.03 to 0.14) | 0.09 (0.06, -0.02 to 0.20) |
|  | **b (*p*)** |  | 0.04 (*p*=0.57) | 0.01 (*p*=0.87) | 0.09 (*p*=0.19) | 0.11 (*p*=0.10) |
|  | **B (SE, 95% CI)** | **Psychological risk** | 0.92 (0.90, -0.86 to 2.69) | 3.59 (1.62, 0.41 to 6.77)* | 3.20 (1.08, 1.07 to 5.33)* | 4.48 (1.40, 1.72 to 7.24)* |
|  | **b (*p*)** |  | 0.18 (*p*=0.31) | 0.36 (*p*=0.03)* | 0.48 (*p*=0.00)* | 0.52 (*p*=0.00)* |
|  | **B (SE, 95% CI)** | **Relationship building * Psychological risk** | 0.06 (0.06, -0.06 to 0.17) | 0.07 (0.10, -0.13 to 0.28) | -0.01 (0.07, -0.15 to 0.12) | -0.03 (0.09, -0.21 to 0.14) |
|  | **b (*p*)** |  | 0.17 (*p*=0.32) | 0.12 (*p*=0.47) | -0.03 (*p*=0.86) | -0.06 (*p*=0.73) |
|  | ***R-square change*** |  | *0.00 (p=0.32)* | *0.00 (p=0.47)* | *0.00 (p=0.86)* | *0.00 (p=0.73)* |
|  | **B (SE, 95% CI)** | **Generic** | -0.08 (0.03, -0.14 to -0.02)* | -0.16 (0.05, -0.26 to 0=0.05)* | -0.03 (0.04, -0.10 to 0.04) | 0.01 (0.05, -0.08 to 0.10) |
|  | **b (*p*)** |  | -0.18 (*p*=0.01)* | -0.18 (*p*=0.00)* | -0.05 (*p*=0.44) | 0.01 (*p*=0.83) |
|  | **B (SE, 95% CI)** | **Psychological risk** | 1.03 (0.63, -0.22 to 2.27) | 3.65 (1.13, 1.43 to 5.87)* | 2.29 (0.77, 0.78 to 3.79)* | 3.70 (1.00, 1.74 to 5.65) |
|  | **b (*p*)** |  | 0.20 (*p*=0.11) | 0.37 (*p*=0.00)* | 0.35 (*p*=0.00)* | 0.43 (*p*<0.05) |
|  | **B (SE, 95% CI)** | **Generic * Psychological risk** | 0.05 (0.05, -0.05 to 0.15) | 0.07 (0.09, -0.12 to 0.25) | 0.06 (0.06, -0.07 to 0.18) | 0.02 (0.08, -0.14 to 0.18) |
|  | **b (*p*)** |  | 0.12 (*p*=0.34) | 0.08 (*p*=0.47) | 0.11 (*p*=0.36) | 0.03 (*p*=0.81) |
|  | ***R-square change*** |  | *0.00 (0.34)* | *0.00 (p=0.47)* | *0.00 (p=0.36)* | *0.00 (p=0.81)* |
|  | **B (SE, 95% CI)** | **Cognitive** | 0.06 (0.03, -0.01 to 0.12) | -0.01 (0.06, -0.13 to 0.10) | 0.04 (0.04, -0.04 to 0.11) | 0.08 (0.05, -0.02 to 0.18) |
|  | **b (*p*)** |  | 0.12 (*p*=0.08) | -0.02 (*p*=0.82) | 0.06 (*p*=0.36) | 0.10 (*p*=0.12) |
|  | **B (SE, 95% CI)** | **Psychological risk** | 0.94 (0.78, -0.59 to 2.47) | 3.31 (1.40, 0.55 to 6.07)* | 2.92 (0.94, 1.07 to 4.78)* | 3.83 (1.22, 1.43 to 6.23)* |
|  | **b (*p*)** |  | 0.18 (*p*=0.23) | 0.33 (*p*=0.02)* | 0.44 (*p*=0.00)* | 0.45 (*p*=0.00)* |
|  | **B (SE, 95% CI)** | **Cognitive * Psychological risk** | 0.06 (0.05, -0.04 to 0.17) | 0.10 (0.10, -0.09 to 0.29) | 0.00 (0.07, -0.13 to 0.13) | 0.01 (0.08, -0.16 to 0.18) |
|  | **b (*p*)** |  | 0.17 (*p*=0.26) | 0.15 (*p*=0.31) | 0.01 (*p*=0.96) | 0.02 (*p*=0.91) |
|  | ***R-square change*** |  | *0.00 (p=0.26)* | *0.00 (p=0.31)* | *0.00 (p=0.96)* | *0.00 (p=0.91)* |

**p<0.05; Red highlight=difference in statistical significance from data reported in paper*
